# Supplementary material for: Characterization of FLOWERING LOCUS T‐related genes and their putative gene regulatory network in semi‐winter Brassica napus cultivar Zhongshaung11
Source: Plant J. 2025 Aug 26;123(4):e70443. doi: 10.1111/tpj.70443 (PMC12380478; doi:10.1111/tpj.70443)
Supplement: Supplementary file 4 — Figure S1. Gene structure of FT homologous genes in B. napus. Figure S2. Synteny analysis of FT in A. thaliana against B. napus ZS11 genomic background. Figure S3. (a) and (b) Flowering time analysis of independent T2 lines. Figure S4. Alignment of FT‐like genes from B. napus and A. thaliana. Figure S5. Alignment of BnaFT.C6 from different accessions. Figure S6. Genome structure alignment analysis. Figure S7. Phylogenetic tree constructed from the alignment of NF‐Y homologs in A. thaliana and B. napus. Table S1. Last results of FT against proteins predicted in B. napus ZS11. Table S2. NF‐Y genes in Brassica napus ZS11 and A. thaliana. Table S3. Oligonucleotide sequences used in this study. Table S4. Summary Table of FT like genes in A. thaliana, B. napus var. ZS11 and Darmor, S. parvula, B. rapa and B.oleracea. [file TPJ-123-0-s001.pdf]

Supplemental Figures and Tables

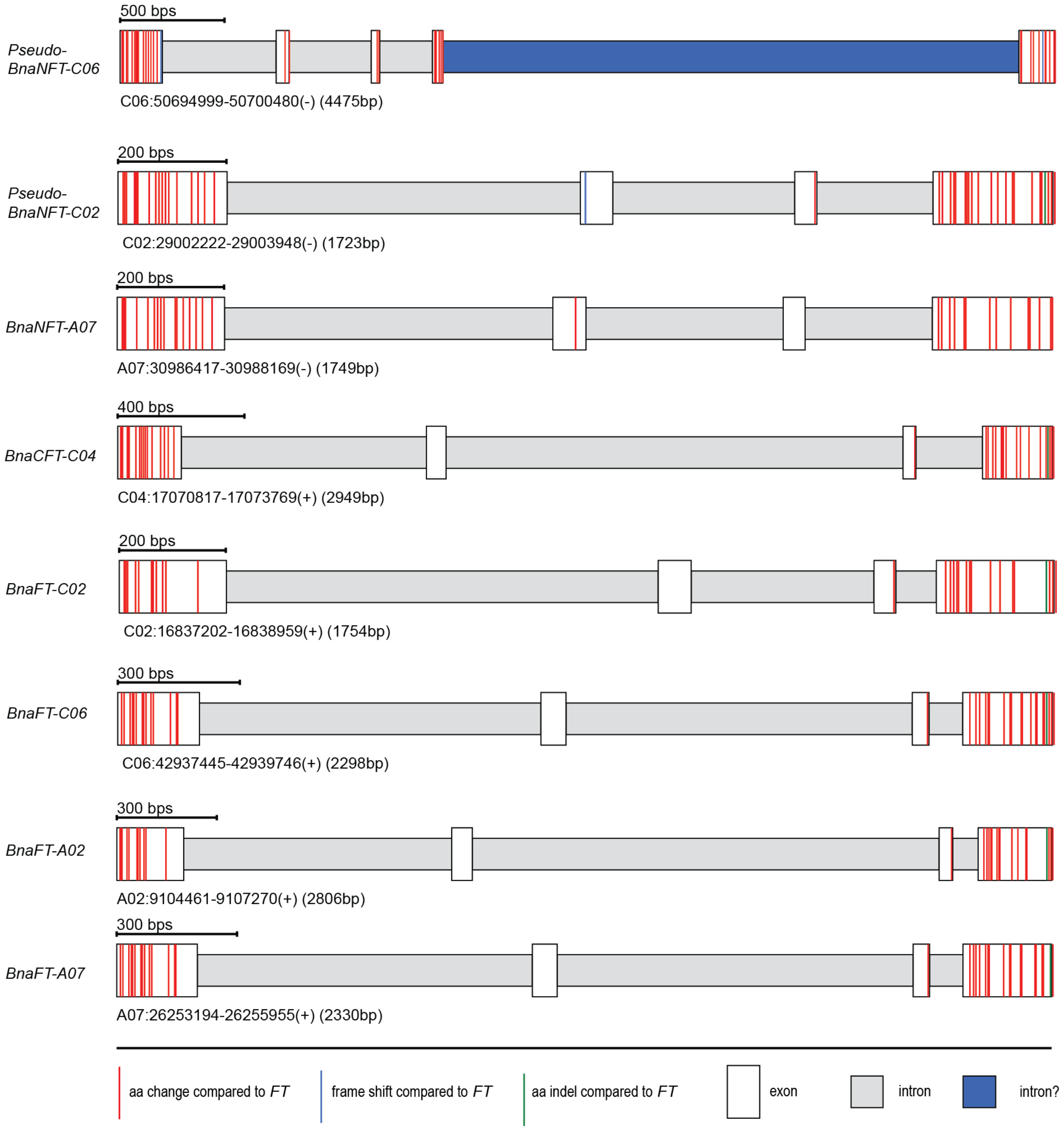

Supplemental Figure 1. Gene structure of *FT* homologous genes in *B. napus*. Boxes indicate protein coding regions, red lines indicate aa changes, blue lines frame shifts and green lines in-frame amino-acid indels compared to FT.

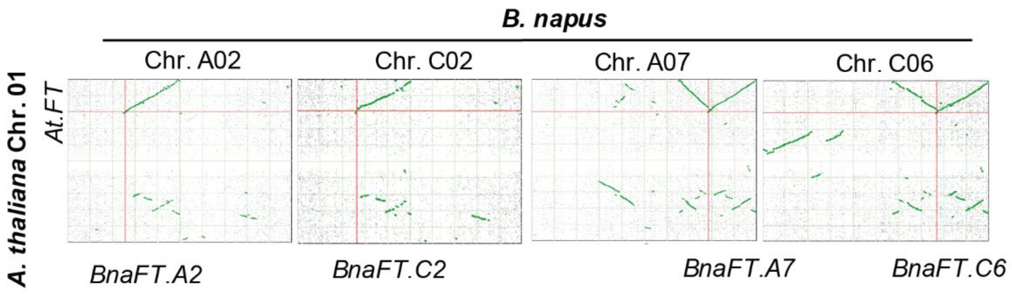

Supplemental Figure 2. Synteny analysis of *FT* in *A. thaliana* against *B. napus* ZS11 genomic background.

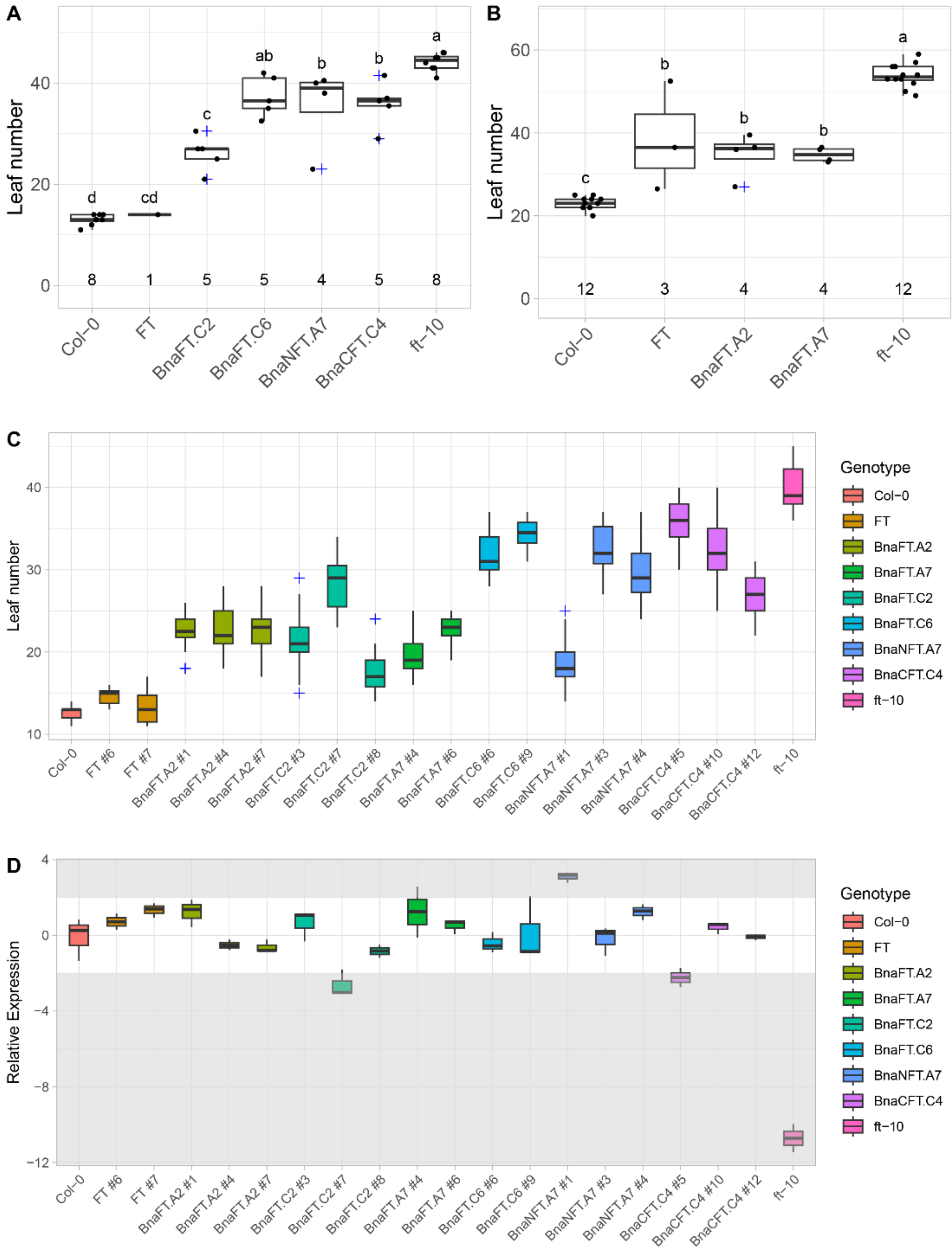

Supplemental Figure 3. (A) and (B) Flowering time analysis of independent T2 lines. Flowering time is measured as total leaf number from segregating independent T2 lines grown in LD at 22°C in greenhouse conditions. Values of at least 10 plants from segregating lines were collapsed to their median. Statistical testing was performed by one-factor ANOVA followed by HSD, letters indicate confidence intervals. The number of independent lines is indicated above the category axis within each graph. **(C)** Flowering time analysis of T3 lines from T2 lines showing 3:1 segregation in A and B. **(D)** Analysis of transgene expression in individual T3 lines analysed in (C). Samples for RNA extraction were collected at ZT16 from three biological replicates of 14-day old seedlings grown in LD at 22°C in greenhouse conditions. Expression data were titrated against the plasmids used for transformation using a primer pair against the BAR marker gene to correct for dilution differences. PP2A was used as housekeeping gene, all data were related to the mean of FT expression in Col-0. The white area indicates a band within +/-log2-fold change of 2. Within this area, flowering time appears mostly uncorrelated to expression level, while outliers in the grey shaded area show a corresponding flowering response.

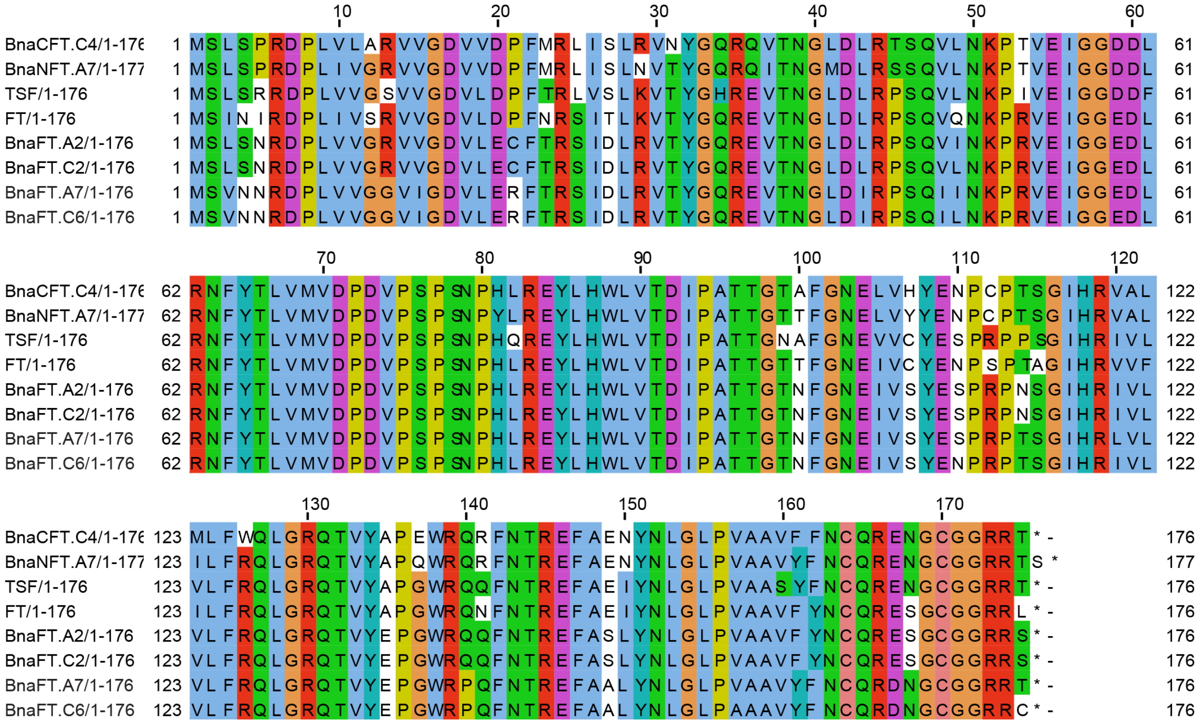

Supplemental Figure 4. Alignment of FT-like genes from *B. napus* and *A. thaliana*.

Similar colours indicate conserved amino-acids with similar biochemical properties.

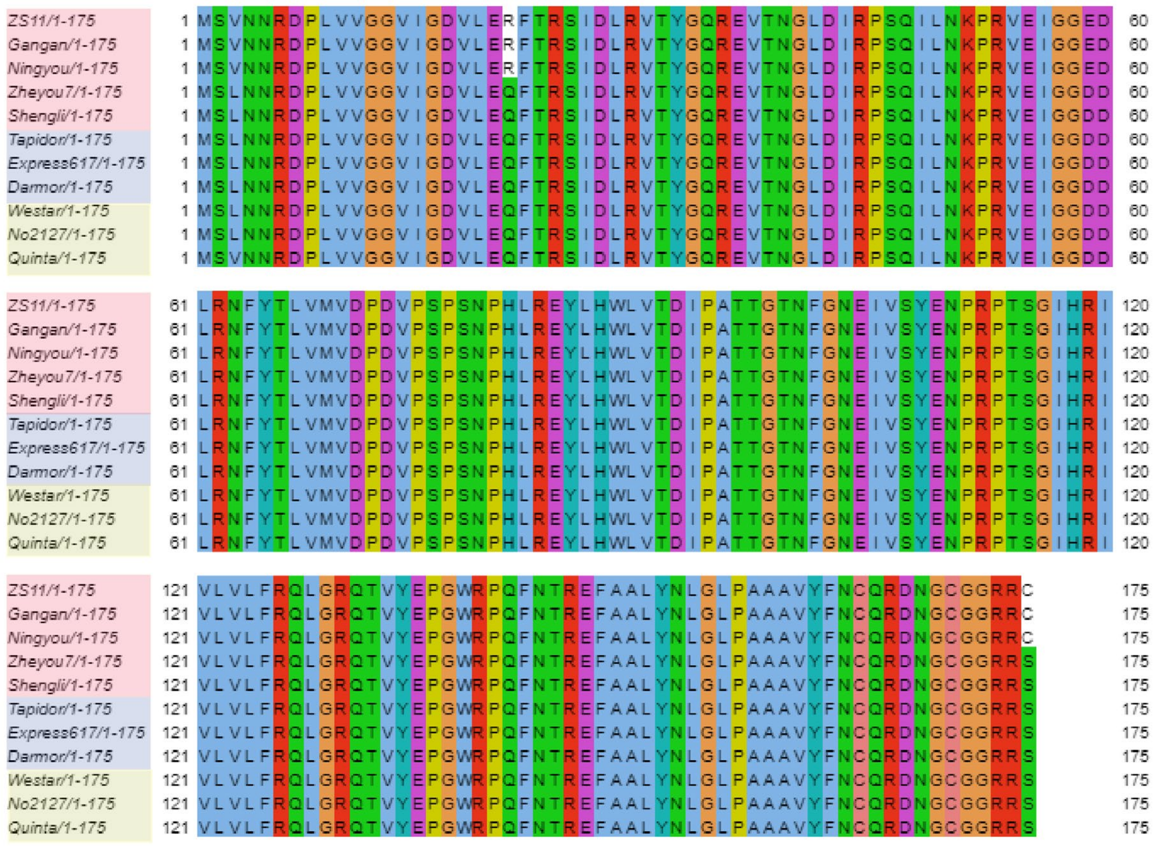

Supplemental Figure 5. Alignment of BnaFT.C6 from different accessions. Coloured boxes in indicate semi-winter (red), winter (blue) and spring (yellow) cultivars.

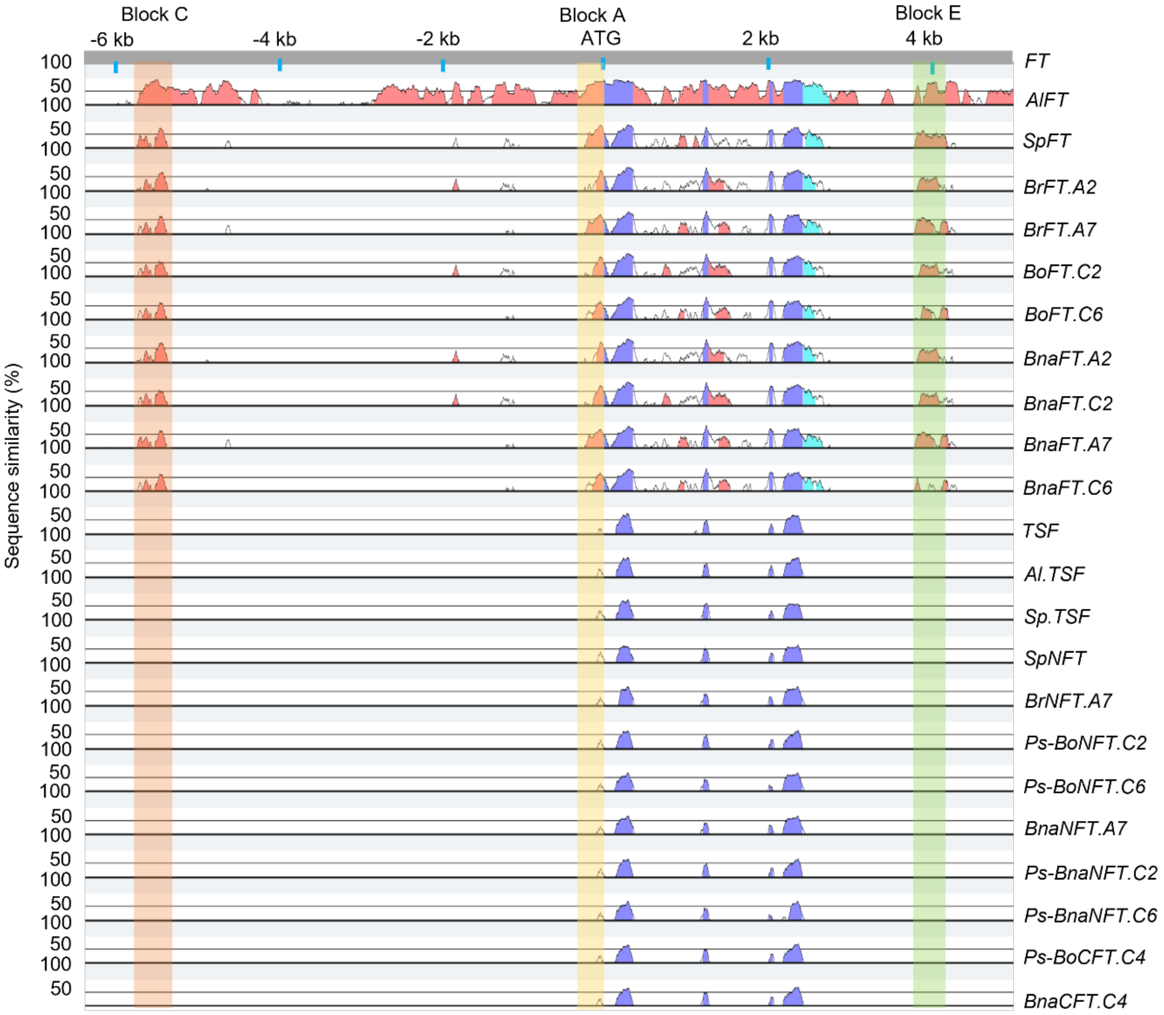

Supplemental Figure 6. Genome structure alignment analysis.

Pairwise alignment of genomic sequences of *FT*, *TSF*, *NFT*, *CFT* homologs from different species to the *FT* genomic sequence using mVISTA. The graphical output shows base-pair identity in sliding 100-bp windows in a range of 50% to 100%. The pink regions are "Conserved Non-Coding Sequences" ("CNS"), the dark blue regions are exons, and the light-blue regions are UTRs. Orange, yellow and green boxes indicate the location of conserved Block C, Block A, and Block E, respectively.

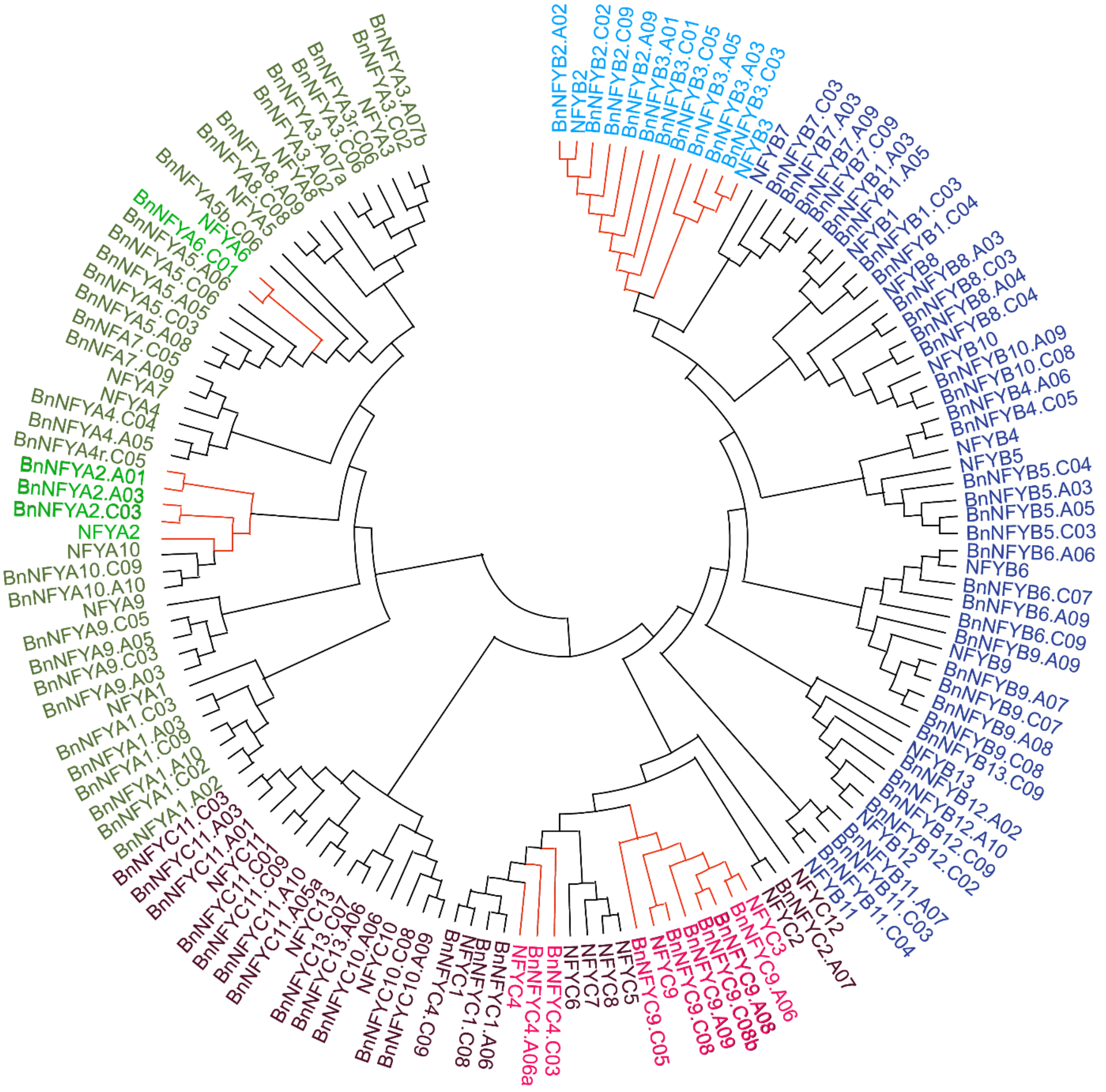

Supplemental Figure 7. Phylogenetic tree constructed from the alignment of NF-Y homologs in *A. thaliana* and *B. napus*. NF-YA, NF-YB and NF-YC homologs are coloured in green, blue and red, respectively. Branches marked in red indicate *A. thaliana* NF-Y proteins involved in *FT* regulation and their orthologs in *B. napus*.

Query ID	Subject ID	Identity	Alignment Length	Mis-matches	Gap Opening	Query Start	Query End	Subject Start	Subject End	E-value	Bit Score
AT1G65480.1	BnaA02T0156900ZS	**87.69**	**528**	65	0	1	528	1	528	5.30E-191	631
AT1G65480.1	BnaC02T0200600ZS	**87.5**	**528**	66	0	1	528	1	528	7.70E-190	628
AT1G65480.1	BnaA07T0282700ZS	**86.74**	**528**	70	0	1	528	1	528	3.30E-185	615
AT1G65480.1	BnaC06T0323800ZS	**86.55**	**528**	71	0	1	528	1	528	4.70E-184	612
AT1G65480.1	BnaA07T0365100ZS	**82.92**	**521**	89	0	1	521	1	521	1.20E-159	544
AT1G65480.1	BnaC04T0181400ZS	**81.23**	**522**	98	0	1	522	1	522	3.00E-150	517
AT1G65480.1	BnaC02T0302200ZS	**81.61**	310	57	0	213	522	294	603	5.00E-84	311
AT1G65480.1	BnaC06T0428800ZS	**81.48**	270	49	1	1	269	1	270	3.90E-68	259
AT1G65480.1	BnaC02T0302200ZS	77.61	201	45	0	1	201	1	201	3.40E-43	177
AT1G65480.1	BnaA06T0273500ZS	63.36	393	141	1	15	404	12	404	5.70E-37	157
AT1G65480.1	BnaC03T0559000ZS	62.85	393	143	1	15	404	12	404	4.70E-35	150
AT1G65480.1	BnaA03T0012400ZS	60.08	491	193	1	18	505	27	517	4.30E-34	147
AT1G65480.1	BnaC03T0016500ZS	59.47	491	196	1	18	505	18	508	3.20E-31	138

Supplemental Table 1. Last results of FT against proteins predicted in *B. napus* ZS11. Identities in bold were over the threshold to consider FT like proteins. Alignment Length in bold distinguish FT like proteins from fragments encoded by pseudogenes.

Gene	Alias	Synteny Group	Genome	family	species
AT5G12840	NFYA1	A	At	NFYA	*A.thaliana*
BnaA02G0045100ZS	BnaNFYA1.A02	A	A	NFYA	*B.napus*
BnaA03G0050200ZS	BnaNFYA1.A03	A	A	NFYA	*B.napus*
BnaA10G0226800ZS	BnaNFYA1.A10	A	A	NFYA	*B.napus*
BnaC02G0051800ZS	BnaNFYA1.C02	A	C	NFYA	*B.napus*
BnaC03G0058100ZS	BnaNFYA1.C03	A	C	NFYA	*B.napus*
BnaC09G0532200ZS	BnaNFYA1.C09	A	C	NFYA	*B.napus*
AT3G05690	NFYA2	B	At	NFYA	*A.thaliana*
BnaA01G0396200ZS	BnaNFYA2.A01	B	A	NFYA	*B.napus*
BnaA03G0300100ZS	BnaNFYA2.A03	B	A	NFYA	*B.napus*
BnaC03G0359700ZS	BnaNFYA2.C03	B	C	NFYA	*B.napus*
BnaC05G0533300ZS	BnaNFYA2.C05	B	C	NFYA	*B.napus*
AT1G72830	NFYA3	C	At	NFYA	*A.thaliana*
BnaA02G0203300ZS	BnaNFYA3.A02	C	A	NFYA	*B.napus*
BnaA07G0257900ZS	BnaNFYA3.A07b	C	A	NFYA	*B.napus*
BnaA07G0335600ZS	BnaNFYA3.A07a	C	A	NFYA	*B.napus*
BnaC02G0270200ZS	BnaNFYA3.C02	C	C	NFYA	*B.napus*
BnaC06G0394200ZS	BnaNFYA3.C06	C	C	NFYA	*B.napus*
BnaC06G0394000ZS	BnaNFYA3d.C06	C	C	NFYA	*B.napus*
AT2G34720	NFYA4	D	At	NFYA	*A.thaliana*
BnaA05G0097800ZS	BnaNFYA4.A05	D	A	NFYA	*B.napus*
BnaC04G0121000ZS	BnaNFYA4.C04	D	C	NFYA	*B.napus*
AT1G54160	NFYA5	E	At	NFYA	*A.thaliana*
BnaA05G0156300ZS	BnaNFYA5.A05	E	A	NFYA	*B.napus*
BnaA06G0007400ZS	BnaNFYA5.A06	E	A	NFYA	*B.napus*
BnaA08G0010100ZS	BnaNFYA5.A08	E	A	NFYA	*B.napus*
BnaC03G0804900ZS	BnaNFYA5.C03	E	C	NFYA	*B.napus*
BnaC06G0084400ZS	BnaNFYA5b.C06	E	C	NFYA	*B.napus*
BnaC06G0136700ZS	BnaNFYA5.C06	E	C	NFYA	*B.napus*
AT3G14020	NFYA6	F	At	NFYA	*A.thaliana*
BnaC01G0442400ZS	BnaNFYA6.C01	F	C	NFYA	*B.napus*
AT1G30500	NFYA7	G	At	NFYA	*A.thaliana*
BnaA09G0409000ZS	BnaNFA7.A09	G	A	NFYA	*B.napus*
BnaC05G0264300ZS	BnaNFA7.C05	G	C	NFYA	*B.napus*
AT1G17590	NFYA8	H	At	NFYA	*A.thaliana*
BnaA09G0616800ZS	BnaNFYA8.A09	H	A	NFYA	*B.napus*
BnaC08G0472300ZS	BnaNFYA8.C08	H	C	NFYA	*B.napus*
AT3G20910	NFYA9	I	At	NFYA	*A.thaliana*
BnaA03G0365100ZS	BnaNFYA9.A03	I	A	NFYA	*B.napus*
BnaA05G0347700ZS	BnaNFYA9.A05	I	A	NFYA	*B.napus*
BnaC03G0445600ZS	BnaNFYA9.C03	I	C	NFYA	*B.napus*
BnaC05G0377800ZS	BnaNFYA9.C05	I	C	NFYA	*B.napus*
AT5G06510	NFYA10	J	At	NFYA	*A.thaliana*
BnaA10G0267400ZS	BnaNFYA10.A10	J	A	NFYA	*B.napus*

BnaC09G0583300ZS	BnaNFYA10.C09	J	C	NFYA	*B.napus*
AT2G38880	NFYB1	A	At	NFYB	*A.thaliana*
BnaA03G0188600ZS	BnaNFYB1.A03	A	A	NFYB	*B.napus*
BnaA05G0068900ZS	BnaNFYB1.A05	A	A	NFYB	*B.napus*
BnaC03G0221700ZS	BnaNFYB1.C03	A	C	NFYB	*B.napus*
BnaC04G0077900ZS	BnaNFYB1.C04	A	C	NFYB	*B.napus*
AT5G47640	NFYB2	B	At	NFYB	*A.thaliana*
AT5G47670	NFYB6	B	At	NFYB	*A.thaliana*
BnaA02G0309400ZS	BnaNFYB2.A02	B	A	NFYB	*B.napus*
BnaA09G0215400ZS	BnaNFYB2.A09	B	A	NFYB	*B.napus*
BnaA09G0215600ZS	BnaNFYB6.A09	B	A	NFYB	*B.napus*
BnaC02G0419600ZS	BnaNFYB2.C02	B	C	NFYB	*B.napus*
BnaC07G0261700ZS	BnaNFYB6.C07	B	C	NFYB	*B.napus*
BnaC09G0250000ZS	BnaNFYB2.C09	B	C	NFYB	*B.napus*
BnaC09G0250300ZS	BnaNFYB6.C09	B	C	NFYB	*B.napus*
BnaA06G0414700ZS	BnaNFYB6.A06	B	A	NFYB	*B.napus*
AT4G14540	NFYB3	C	At	NFYB	*A.thaliana*
BnaA01G0344800ZS	BnaNFYB3.A01	C	At	NFYB	*B.napus*
BnaA03G0343900ZS	BnaNFYB3.A03	C	A	NFYB	*B.napus*
BnaA05G0394900ZS	BnaNFYB3.A05	C	A	NFYB	*B.napus*
BnaC01G0426200ZS	BnaNFYB3.C01	C	C	NFYB	*B.napus*
BnaC03G0414900ZS	BnaNFYB3.C03	C	C	NFYB	*B.napus*
BnaC05G0441600ZS	BnaNFYB3.C05	C	C	NFYB	*B.napus*
AT1G09030	NFYB4	D	At	NFYB	*A.thaliana*
BnaA06G0054400ZS	BnaNFYB4.A06	D	A	NFYB	*B.napus*
BnaC05G0067400ZS	BnaNFYB4.C05	D	C	NFYB	*B.napus*
AT2G47810	NFYB5	E	At	NFYB	*A.thaliana*
BnaA03G0228800ZS	BnaNFYB5.A03	E	A	NFYB	*B.napus*
BnaA05G0001800ZS	BnaNFYB5.A05	E	A	NFYB	*B.napus*
BnaC03G0269200ZS	BnaNFYB5.C03	E	C	NFYB	*B.napus*
BnaC04G0002000ZS	BnaNFYB5.C04	E	C	NFYB	*B.napus*
AT2G13570	NFYB7	F	At	NFYB	*A.thaliana*
BnaA03G0391200ZS	BnaNFYB7.A03	F	A	NFYB	*B.napus*
BnaA09G0104400ZS	BnaNFYB7.A09	F	A	NFYB	*B.napus*
BnaC03G0484300ZS	BnaNFYB7.C03	F	C	NFYB	*B.napus*
BnaC09G0105300ZS	BnaNFYB7.C09	F	C	NFYB	*B.napus*
AT2G37060	NFYB8	G	At	NFYB	*A.thaliana*
BnaA03G0177300ZS	BnaNFYB8.A03	G	A	NFYB	*B.napus*
BnaA04G0237700ZS	BnaNFYB8.A04	G	A	NFYB	*B.napus*
BnaC03G0207500ZS	BnaNFYB8.C03	G	C	NFYB	*B.napus*
BnaC04G0553200ZS	BnaNFYB8.C04	G	C	NFYB	*B.napus*
AT1G21970	NFYB9	H	At	NFYB	*A.thaliana*
BnaA07G0126300ZS	BnaNFYB9.A07	H	A	NFYB	*B.napus*
BnaA08G0241300ZS	BnaNFYB9.A08	H	A	NFYB	*B.napus*
BnaA09G0465400ZS	BnaNFYB9.A09	H	A	NFYB	*B.napus*

BnaC07G0185300ZS	BnaNFYB9.C07	H	C	NFYB	*B.napus*
BnaC08G0270100ZS	BnaNFYB9.C08	H	C	NFYB	*B.napus*
AT3G53340	NFYB10	I	At	NFYB	*A.thaliana*
BnaA09G0495300ZS	BnaNFYB10.A09	I	A	NFYB	*B.napus*
BnaC08G0334000ZS	BnaNFYB10.C08	I	C	NFYB	*B.napus*
AT2G27470	NFYB11	J	At	NFYB	*A.thaliana*
BnaA07G0155000ZS	BnaNFYB11.A07	J	A	NFYB	*B.napus*
BnaC03G0276400ZS	BnaNFYB11.C03	J	C	NFYB	*B.napus*
BnaC04G0206800ZS	BnaNFYB11.C04	J	C	NFYB	*B.napus*
AT5G08190	NFYB12	K	At	NFYB	*A.thaliana*
BnaA02G0028200ZS	BnaNFYB12.A02	K	A	NFYB	*B.napus*
BnaA10G0255300ZS	BnaNFYB12.A10	K	A	NFYB	*B.napus*
BnaC02G0030900ZS	BnaNFYB12.C02	K	C	NFYB	*B.napus*
BnaC09G0569100ZS	BnaNFYB12.C09	K	C	NFYB	*B.napus*
AT5G23090	NFYB13	L	At	NFYB	*A.thaliana*
BnaC09G0063500ZS	BnaNFYB13.C09	L	C	NFYB	*B.napus*
AT3G48590	NFYC1	A	At	NFYC	*A.thaliana*
BnaA06G0165700ZS	BnaNFYC1.A06	A	A	NFYC	*B.napus*
BnaC08G0290400ZS	BnaNFYC1.C08	A	C	NFYC	*B.napus*
AT1G56170	NFYC2	B	At	NFYC	*A.thaliana*
BnaA07G0203700ZS	BnaNFYC2.A07	B2	A	NFYC	*B.napus*
AT1G54830	NFYC3	C	At	NFYC	*A.thaliana*
AT5G63470	NFYC4	D	At	NFYC	*A.thaliana*
BnaA06G0286000ZS	BnaNFYC4.A06a	D	A	NFYC	*B.napus*
BnaC03G0542200ZS	BnaNFYC4.C03	D	C	NFYC	*B.napus*
BnaC09G0077900ZS	BnaNFYC4.C09	D	C	NFYC	*B.napus*
AT5G50470	NFYC7	E	At	NFYC	*A.thaliana*
AT5G50480	NFYC6	E	At	NFYC	*A.thaliana*
AT5G50490	NFYC5	E	At	NFYC	*A.thaliana*
AT5G27910	NFYC8	F	At	NFYC	*A.thaliana*
AT1G08970	NFYC9	G	At	NFYC	*A.thaliana*
BnaA06G0054000ZS	BnaNFYC9.A06	G	A	NFYC	*B.napus*
BnaA08G0297000ZS	BnaNFYC9.A08	G	A	NFYC	*B.napus*
BnaA09G0663800ZS	BnaNFYC9.A09	G	A	NFYC	*B.napus*
BnaC05G0066900ZS	BnaNFYC9.C05	G	C	NFYC	*B.napus*
BnaC08G0189800ZS	BnaNFYC9.C08b	G	C	NFYC	*B.napus*
BnaC08G0528300ZS	BnaNFYC9.C08	G	C	NFYC	*B.napus*
AT1G07980	NFYC10	H	At	NFYC	*A.thaliana*
BnaA06G0046600ZS	BnaNFYC10.A06	H	A	NFYC	*B.napus*
BnaA09G0668200ZS	BnaNFYC10.A09	H	A	NFYC	*B.napus*
BnaC08G0533200ZS	BnaNFYC10.C08	H	C	NFYC	*B.napus*
AT3G12480	NFYC11	I	At	NFYC	*A.thaliana*
BnaA01G0367300ZS	BnaNFYC11.A01	I	A	NFYC	*B.napus*
BnaA03G0328100ZS	BnaNFYC11.A03	I	A	NFYC	*B.napus*
BnaA05G0425500ZS	BnaNFYC11.A05a	I	A	NFYC	*B.napus*

BnaA05Z0425400ZS	BnaNFYC11.A05b	I	A	NFYC	*B.napus*
BnaC01G0458700ZS	BnaNFYC11.C01	I	C	NFYC	*B.napus*
BnaC03G0393600ZS	BnaNFYC11.C03	I	C	NFYC	*B.napus*
BnaA10G0258900ZS	BnaNFYC11.A10	new	A	NFYC	*B.napus*
BnaC09G0573000ZS	BnaNFYC11.C09	new	C	NFYC	*B.napus*
AT5G38140	NFYC12	J	At	NFYC	*A.thaliana*
AT5G43250	NFYC13	K	At	NFYC	*A.thaliana*
BnaA06G0442500ZS	BnaNFYC13.A06	K	A	NFYC	*B.napus*
BnaC07G0218200ZS	BnaNFYC13.C07	K	C	NFYC	*B.napus*

Supplemental Table 2. NF-Y genes in Brassica napus ZS11 and A. thaliana. Syntenic genes per group are annotated to the same letter.

Application	Primer name	Sequence
qPCR in *B. napus*	BnaFT.A2-qF	GGTATTCATCGTATCGTGCTCG
	BnaFT.A2-qR	CAAGTTATTAAAAGAAGAAGAGGCTC
	BnaFT.C2-qF	GGTATTCATCGTATCGTGCTG
	BnaFT.C2-qR	GTTATTAAAAGAAGAAGAGGCTCATC
	BnaFT.A7-qF	CCACCTCGGGAATTCATCGTC
	BnaFT.A7-qR	CCATGACCCATCGATCTAAG
	BnaFT.C6-qF	CAAACGGTGTATGAACCAGG
	BnaFT.C6-qR	TCTAAGGAAGAAGCCCATCG
	BnaNFT.A7-qF	CGAGAGACCCTCTTATCGTAGG
	BnaNFT.A7-qR	AATCTCAACCGTTGGTTTGTTC
	BnaCFT.C4-qF	CGAGAGATCCTCTTGTGCTTGC
	BnaCFT.C4-qR	GATCTCGACCGTTGGTTTATTT
	BnaCO.A10-qF	ACGTATGGCTCCTCAGGAAGTCAC
	BnaCO.A10-qR	TCTGAATTAGAGGTTCAGGTAGTTTCT
	BnaCO.C09-qF	TAAACAAGACTGCATCGTACCAGAGA
	BnaCO.C09-qR	GTCAGTTTCCATTGATGGATTGTATG
	BnaENTH-qF	GTTTAGACCCGTTGCTGCTC
	BnaENTH-qR	TTGTCCATCTCAGCCATTTG
qPCR in *A.thaliana*	RT-BnFT_cDNA_F	GGTGGAGAAGACCTAAGGAA
	RT-BnFT_cDNA_R1	GGTTCATACACTGTTTGCCT
	RT-BnCFT_cDNA_F	TGTCCCACTTCCGGAATTCA
	RT-BnCFT_cDNA_R	TCCTCCACAGCCATTCTCTC
	RT-BnNFT_cDNA_F	ACGAGAATCCATGTCCCACA
	RT-BnNFT_cDNA_R	TGAAGTAAACAGCAGCCACG
	RT-FT_cDNA_F	GGTGGAGAAGACCTCAGGAA
	RT-FT_cDNA_R	ACCCTGGTGCATACACTGTT
	BASTA_qPCR_F	CCAGTTCCCGTGCTTGAA
	BASTA_qPCR_R	AGAGCGTGGTCGCTGTCAT
	PP2A_qPCR_F	ACACAATTCGTTGCTGTCTTCT
	PP2A_qPCR_R	TGCTTGGTGGAGCTAAGTGA
Complementation with plasmid driven by FT Block (C+A)	BnaFT.A2C2-pFT-F	TGGTGATATCAAGCTTATGTCTTTAAGTAATAGAGATCCTCTTG
	BnaFT.A2-pFT-R	GATCGGGGAAATTCGAGCTCCTAACTTCTTCGTCCTCCG
	BnaFT.C2-pFT-R	GATCGGGGAAATTCGAGCTCCTAACTTCTTCGTCCTCCGCAGC
	BnaFT.A7C6-pFT-F	TGGTGATATCAAGCTTATGTCTGTAAATAACAGAGATCCTCT
	BnaFT.A7C6m-pFT-R	GATCGGGGAAATTCGAGCTCCTAAGTTCTTCGTCCTCCG
	BnaFT.C6C7m-pFT-R	GATCGGGGAAATTCGAGCTCCTAACATCTTCGTCCTCCG
	BnaNFT.A7-pFT-F/	TGGTGATATCAAGCTTATGTCATTAAGTCCGAGAGACCCT
	BnaNFT.A7-pFT-R	GATCGGGGAAATTCGAGCTCCTACGAGGTCCTTCTTCCTCCG
	BnaCFT.C4-pFT-F	TGGTGATATCAAGCTTATGTCTTTAAGTCCGAGAGATCCTC
	BnaCFT.C4-pFT-R	GATCGGGGAAATTCGAGCTCCTATGTTCTTCTTCCTCCACAGCCA
	Plasmid-V-F1	CACAGAGAAACCACCTGTTTGTT
	Plasmid-V-R1	TATGATAATCATCGCAAGACCG
ComplemenComplementation with	BnaFT.A7C6-pFD-F	AGGTGGTGGAAGTTACCCTTACGATGTGCCTGATTACGCTGGAAGTTCTGTAAATAACAGAGATCCTCTTG

plasmid driven by FD promoter	pFD-2HA-F	CTTCTGTTCTCTTTTCCAATGTACCCATACGATGTGCCTGATTACGCTGGA GGTGGTGGAAGTTACCCTTACG
	BnaFT.A7-pFD-R	CAAGGACTTGTAGATTTCCTAAGTTCTTCGTCCTCCG
	BnaFT.C6-pFD-R	CAAGGACTTGTAGATTTCCTAACATCTTCGTCCTCCG
	At.FT-pFD-F	AGGTGGTGGAAGTTACCCTTACGATGTGCCTGATTACGCTGGAAGTTCTA TAAATATAAGAGACCCTCT
	At.FT-pFD-R	CAAGGACTTGTAGATTTCCTAAAGTCTTCTTCCTCCGCA
	Plasmid-V-F2	ACCGGCTAAAGTCAAGAACCTCT
	Plasmid-V-R2	CCGGGTCTTTTGTTTTACATCTTC
	2HA-tag	TACCCATACGATGTGCCTGATTACGCTGGAGGTGGTGGAAGTTACCCTTA CGATGTGCCTGATTACGCT
Tobacco infiltration, reporter vectors construction	pFT-5.7K-F	CGA ATT GGG TAC AGT ACT CCTCTCTTCGAATTACATTCGTATGA
	pFT-5.7K-R	TCG CGT TTC ACC ATG G CTTTGATCTTGAACAAACAGGTGGT
	pA2-12K-P1-F	CGAATTGGGTACAGTACTGCTATCAATAGTAATTCGATTTCTATGAGC
	pA2-12K-P1-R	ACCAGATGATGCCTGCGTCTATG
	pA2-12K-P2-F	GCAGGCATCATCTGGTGAGAAC
	pA2-12K-P2-R	TCGCGTTTCACCATGGCTTTGATCTAAAACAAACAGGTGG
	pA7-12K-P1-F	CGAATTGGGTACAGTACTGTGCTTTAAACTAGTGACCAGGAG
	pA7-12K-P1-R	TCCAAACTTCTTTGCAACAGACAAAGG
	pA7-12K-P2-F	GCAAAGAAGTTTGGATTCACTCAG
	pA7-12K-P2-R	TCGCGTTTCACCATGGCTCTGATCTAAAACAAACAGGTTG
	pC2-21K-P1-F	CGAATTGGGTACAGTACTGCACAAAAGTTACGTTTTGTTACAGC
	pC2-21K-P1-R	CAGAAGACTTCTCCCAACAGAC
	pC2-21K-P2-F	GTTGGGAGAAGTCTTCTGTGTTAG
	pC2-21K-P2-R	TAGACTATGCTGCCCTTAATCTCTTCG
	pC2-21K-P3-F	GGGCAGCATAGTCTAGTTTTGAG
	pC2-21K-P3-R	GTTAATCTTGTGTTTTCTTGTAGTGAATC
	pC2-21K-P4-F	ACAAGAAAACACAAGATTAACGACG
	pC2-21K-P4-R	TCGCGTTTCACCATGGCTTTGATCTAAAACAAACAGGTGG
	pC6-1.8K-F	CGA ATT GGG TAC AGT ACT GAGCCATTAGATTCGTATGATCACG
	pC6-1.8K-R	TCG CGT TTC ACC ATG G CTCTGATCTAAAACAAACAGGTTGTTTC
Tobacco infiltration, effector vectors construction	adScaI-35S-F	CGAATTGGGTACAGTACT GTGGAGCACGACACAC
	adSacI-CO-R	GATCGGGGAAATTCGAGCTC TCAGAATGAAGGAACAATCCC
	adSacI-BnCO.A10/C9-R	GATCGGGGAAATTCGAGCTC TTATTTTTGGCCATAGAATGAAGG
Tobacco infiltration, plasmid verification	V-629-F1	TGAAGCAACTCCTCGAAAAAGC
	V-629-R1	CAATTCCACACAACATACGAGCC
	V-629-F2	GTGCTGCAAGGCGATTAAGTTG
	V-629-R2	ACGAGTGCTTGAGGGAGGTGAC

Supplemental Table 3. Oligonucleotide sequences used in this study

Arabidopsis (TAIR)		Brassica napus			Schrenkiella parvula		Brassica rapa		Brassica oleracea	
Name	Araport 11	Name	Gene ID ZS11 v0.0	Gene ID Darmor	Name	Gene ID v2.2	Name	Gene ID BraZ1	Name	Gene ID 2J.m1
FT	AT1G65480	BnaFT.A2	BnaA02G0156900ZS	BnaA02g12130D			BrFT.A2	A02p18180		
		BnaFT.C2	BnaC02G0200600ZS	BnaC02g45250D					BoFT.C2	BolC02g021840
		BnaFT.A7	BnaA07G0282700ZS	BnaA07g25310D			BrFT.A7	A07p35130		
		BnaFT.C6	BnaC06G0323800ZS	BnaC06g27090D					BoFT.C6	BolC06g036950
NFT	absent	BnaNFT.A7	BnaA07G0365100ZS	BnaA07g33120D	SpNFT	Sp5g32040	BrNFT.A7	A07p44520		
		BnaNFT.C6	BnaC06T0428800ZS	not predicted					BoNFT.C6	BolC06g048270
		BnaNFT.A2	absent	absent			BrNFT.A2	A02p26660		
		BnaNFT.C2	BnaC02T0302200ZS	BnaC02g23820D					BoNFT.C2	BolC02g033320
CFT	absent	BnaCFT.C4	BnaC04G0181400ZS	BnaC04g14850D		absent		absent	BoCFT.C4	BolC04g020210
TSF	AT4G20370	absent	absent	absent	SpTSF	Sp7g18730		absent		absent

Supplemental Table 4. Summary Table of *FT* like genes in *A. thaliana*, *B. napus* var. ZS11 and Darmor, *S. parvul*a, *B. rapa* and *B.oleracea.* Pseudogenes are marked in red.
